# Endocrine disrupter chemicals affect the humoral antimicrobial activities of gilthead seabream males even upon the cease of the exposure

**DOI:** 10.1038/s41598-020-64522-2

**Published:** 2020-05-14

**Authors:** Yulema Valero, Amanda E. López-Cánovas, M. Carmen Rodenas, Isabel Cabas, Pilar García-Hernández, Marta Arizcun, Alfonsa García-Ayala, Elena Chaves-Pozo

**Affiliations:** 1 Oceanographic Center of Murcia, Spanish Institute of Oceanography (IEO), Carretera de la Azohía s/n. 30860, Puerto de Mazarrón, Murcia, Spain; 2 Department of Cell Biology and Histology, Faculty of Biology, University of Murcia, 30100 Murcia, Spain

**Keywords:** Toxicology, Immunology, Zoology

## Abstract

17α-ethynilestradiol (EE_2_) and tamoxifen (Tmx) are pollutants world-wide distributed in aquatic environments. Gilthead seabream, *Sparus aurata* L., is highlighted as a species model of intensively culture in anthropogenic disturbed environments. The effects of these pollutants on gilthead seabream reproduction and some immune responses have been described but, the humoral innate antimicrobial activities have never received attention. In this work we analysed the latest in the plasma of gilthead seabream males of different ages and reproductive stages treated with 0, 2.5, 5 or 50 μg EE_2_ or 100 μg Tmx g^−1^ food during different times of exposure and of reverting to commercial diet (recovery). The peroxidase and protease activities decreased as the spermatogenesis of the first reproductive cycle (RC) proceeded in control fish. However, only protease and antiprotease activities showed different level at different stages of the second RC in control fish, but showed scarce disruption in fish treated with EE_2_ or Tmx. Peroxidase and bactericide activities are more sensitive to EE_2_, than to Tmx. The effects induced by EE_2_ varied depending on the activity analyzed, the dose and the time of exposure and the reproductive stage and the age of the specimens.

## Introduction

Nowadays, clean waters, with low levels of anthropogenic compounds, are reduced to limited locations scarcely inhabited all over the world (http://www.oceanhealthindex.org/region-scores/maps). The raising use of pharmacological compounds and their presence in the surface water have increased the concern about the unpredictable effects in aquatic organisms and human health. The 17α-ethinylestradiol (EE_2_) is highly estrogenic and widely used in oral contraceptive treatments and hormone therapies and it is known to be in detectable levels in water resources worldwide^1^. By its side, tamoxifen (Tmx) is frequently used in breast cancer therapies^1–3^ and its presence in Mediterranean water effluent sources, it representing a potential ecotoxicological risk^4^. The estrogenic pharmacological compounds are globally distributed in a wide range of aquatic (fresh, estuarine and marine) environments and differences in their disrupting potential have been related to salinity^5^. In fact, their effects are more evident at lower doses in freshwater than in marine water^5^. So, further studies on marine fish species are mandatory to really assess the potential effect of endocrine disrupting chemicals (EDCs) on marine population and aquaculture production. Recently, European Union included EE_2_ together with 17β-estradiol (E_2_) in the European monitoring list of EDCs^6^. Although the levels of EDCs in European marine environments are usually below the environmentally quality standard (100 ng/L), there are hot points with excessively high levels^7,8^. The presence of EE_2_ and Tmx is related to the hardships found in residual water treatment plants to clear them, remaining in water until the end of the process and being released to the fluent waters^9^. In addition, the resistance of synthetic estrogens to degradation and its predicted bioaccumulation throughout the food chain, as it has been suggested using some food-web models^10^, should not be underestimated.

Both compounds, EE_2_ and Tmx, are considered EDCs as they mimic estrogens, binding estrogen receptors (ER) and changing the normal hormone binding to them causing alterations in their hormonal pathways down-stream^3^. In contrast to EE_2,_ Tmx has estrogenic or anti-estrogenic effects depending on the tissue in mammals^11^. In fish, however, Tmx has estrogenic effects but also increased the androgen level and the expression of male related genes such as the double sex-and mab3-related transcription factor 1, *dmrt1*^12^. As matter of fact, fish exposed to EE_2_ and/or Tmx through food intake display alterations in molecular markers of endocrine disruption^12–14^. In fact the concentrations of EE_2_ used in this study are known to ensure effects on some reproductive events in gilthead seabream males^15,16^. The concentration of Tmx assures Tmx-ER interaction and reproductive effect as described previously^12^. In fish as in mammals, both EE_2_ and Tmx strongly affect reproduction and even acute exposure during development might affect the mature reproductive system^12,15–19^. Far beyond reproduction, sex hormones also modulate several biological processes. It is demonstrated in fish that immunity is regulated by estrogens throughout nuclear or membrane receptor mechanisms (see reviews in^20–22^). In fact, several immune responses of fish (both innate and adaptive) are affected by EDCs^2,3^.

The gilthead seabream (*Sparus aurata* L.) is the most relevant species in economic terms in Mediterranean aquaculture^23^, so the effect of these compounds on its physiology are of special relevance. The gilthead seabream is a seasonal breeding protandrous hermaphroditic teleost with a bisexual gonad which develops as male during the first two reproductive cycles (RCs) and then the 40% approximately of the population change to female at the beginning of the third RC^24,25^. The reproductive cycle, during the male phase, is divided into four stages: spermatogenesis, spawning, post- spawning and resting or testicular involution in the first or second RC, respectively, in which the testis undergoes abrupt morphological changes^24^. The sensitivity to EE_2_ has been described to be different in the gonad of gilthead seabream at pre-spermatogenesis and spermatogenesis stages of the first reproductive cycle^16^.

Regarding the potential effects of EDCs on the immune system in gilthead seabream, the nuclear ERα is present in head kidney macrophages and lymphocytes^26^ and the G protein-coupled estrogen receptor (GPER), a membrane estrogen receptor which binds E_2_ and other different estrogenic compounds, has been identified in head kidney acidophilic granulocytes^27^. Interestingly, the nuclear ERβ is expressed in macrophages stimulated either with E_2_ or with bacterial DNA^26,28^. In addition, the production of reactive oxygen intermediates (ROIs), one of the main cell-mediate innate activities, is inhibited by E_2_ on gilthead seabream phagocytes^29^. However, EE_2_ does not affect the naïve cell-mediate innate activities, but alters the fish capacity to respond to infections of both cell-mediated innate and humoral adaptive responses^2,30,31^. Moreover, both compounds, EE_2_ and Tmx, disrupt the response triggered by an immune stimuli as the expression of pro-inflammatory cytokines coding genes, the ROIs production by phagocytes, the percentage of positive immunoglobulin M (IgM^+^) cells and the number of granulocytes in the head kidney upon a challenge^2,30,31^. These effects disappear when the treatment ceases^2,31^. Estrogens also promote leukocyte infiltration in several tissues of gilthead seabream, such as the peritoneal cavity and the gonad, enhancing the ability of endothelial cells to adhere leukocytes^32,33^. From all this studies, it is clear that EDCs affect cell-mediated immunity and humoral adaptive responses in gilthead seabream. The studies that related immune activities with reproductive stages in fish are very scarce as well as the effects of EDCs on serum innate immune activities have poorly been studied in fish, even when the humoral innate activities are key players in the fights against infections in fish.

The aim of this work is to determine if humoral antimicrobial activities differ through the year and/or RC in gilthead seabream and if these activities are differentially altered depending on the reproductive stage in which the specimens are exposed to EDCs. For that, we performed three trials through the first and second RC of gilthead seabream. Thus, (i) trial I try to determine the effect of EE_2_ on the resting stage, previous to the first RC, and on the spermatogenesis stage of the first RC; (ii) Trial II try to determine whether Tmx, as occurs in the reproductive system, has different effect on humoral innate immune response to those of EE_2_ and whether these effects are reversible or not using fish at spermatogenesis stage of the second RC and, (iii) trial III try to determine the effect of long exposure to low doses of EE_2_ and whether these effects are reversible or not starting with fish at spermatogenesis stage of the second RC and finishing at testicular involution stage. As far as we are concerned, E_2_ and EE_2_ can disrupt antibacterial functions in fish^34,35^ but this is the first study dealing with the effect of estrogenic compounds on the humoral innate immune response in gilthead seabream.

## Results

**Figure 1:**
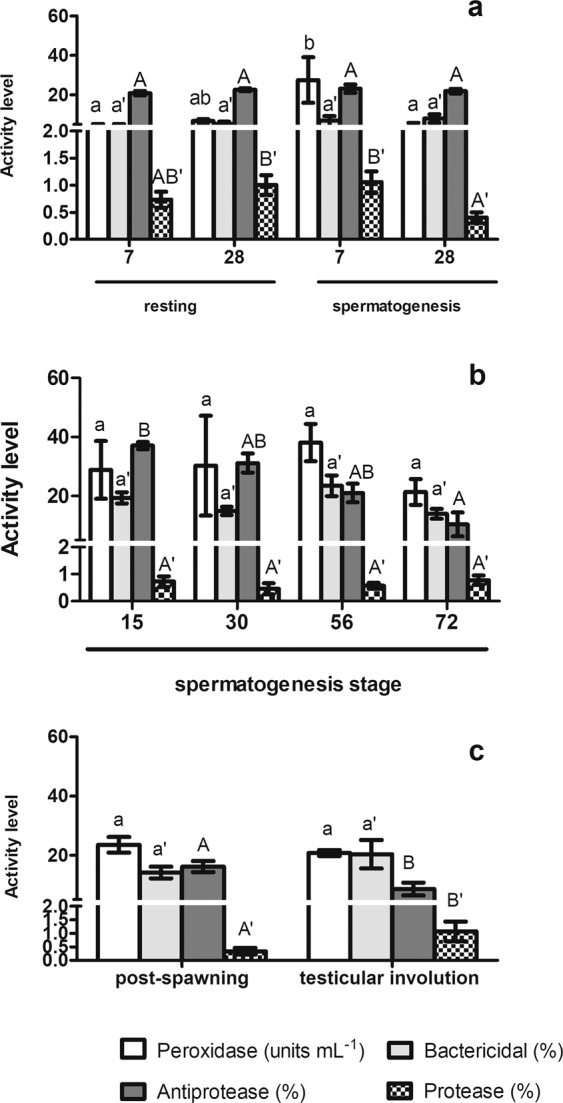
Antimicrobial humoral activities in not treated gilthead seabream males at different times and reproductive stages. Fish at two different moments during the resting stage, previous to the first RC, and the spermatogenesis stage of the first RC (**a**), at different moments during the spermatogenesis stage of the second RC (**b**) and at post-spawning and testicular involution of the second RC (**c**). Data represent means ± standard error (n = 6). Different letters denote statistically significant differences between the groups (P < 0.05).

We first observed that the peroxidase activity level of control fish increased at the beginning of the spermatogenesis stage of the first RC, decreasing later on during this stage to similar levels of the previous resting stage, while the protease activity increased at the beginning of the spermatogenesis stage of the first RC and then decreased reaching lower levels than those reported during the previous resting stage (trial I) (Fig. 1a). During spermatogenesis of the second reproductive cycle, only antiprotease activity level decreased at the end of the stage (from 37.03 ± 1.24% at the beginning to 10.32 ± 2.83% at the end) (Fig. 1b and supplementary data) (trial II). Moreover, antiprotease activity levels decreased even more at testicular involution stage (8.58 ± 2.00%) compared with post-spawning stage levels (16.14 ± 1.69) (Fig. 1c) (trial III). Interestingly, protease activity showed higher levels at testicular involution stage than at post-spawning stage (Fig. 1c) (trial III).

**Figure 2:**
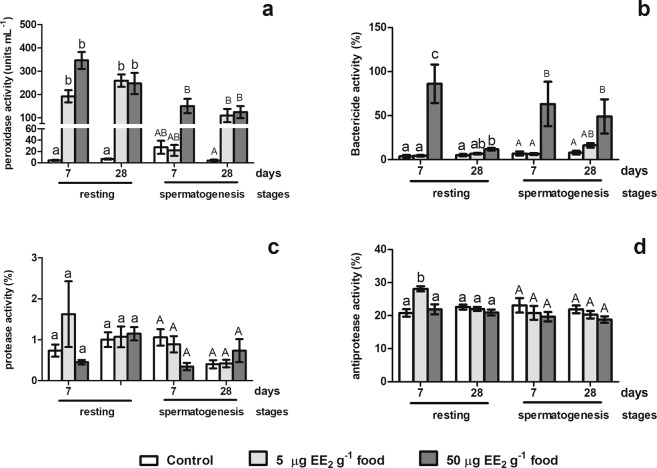
Antimicrobial humoral activities in gilthead seabream males at the resting stage, previous to the first RC, and the spermatogenesis stage of the first RC. The fish were treated with 0, 5 or 50 µg EE_2_ g^−1^ food and sampled at 7 and 28 days of treatment where the peroxidase (**a**), the bactericide (**b**), the protease (**c**) and the antiprotease (**d**) activities were studied. Data represent means ± standard error (n = 6). Different letters denote statistically significant differences between the groups (P < 0.05).

### EE_2_ differently alters the antimicrobial humoral activities in fish at resting and spermatogenesis of the first RC

Our results showed that fish at the resting stage previous of the first RC (R fish) treated with 5 and 50 µg EE_2_ g^−1^ food showed high levels of peroxidase activity at both time point assays (7 and 28 days), while fish at the spermatogenetic stage of the first RC (SG fish) showed an increases of this activity from 7 days onwards with 50 µg of EE_2_ g^−1^ food and after 28 days of 5 µg of EE_2_ g^−1^ food intake (Fig. 2a). Otherwise, in R fish, the bactericidal activity increased 23-fold with the highest dose used (50 µg EE_2_ g^−1^ food) after 7 days but only 2-fold after 28 days. However in SG fish, this activity increased 9- and 6-fold after 7 and 28 days of exposure to 50 µg EE_2_ g^−1^ food, respectively (Fig. 2b). No statistically differences were observed in the protease activity levels neither in R or SG fish (Fig. 2c), while only 5 µg of EE_2_ g^−1^ food intake increased the antiprotease activity levels in R fish, but not in SG fish (Fig. 2d).

**Figure 3:**
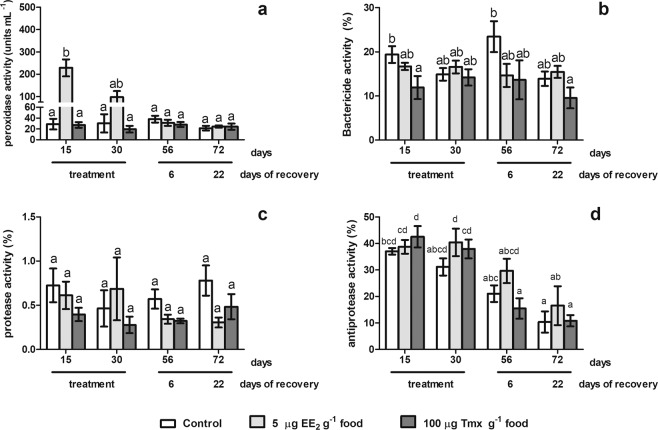
Antimicrobial humoral activities in gilthead seabream males during the spermatogenesis stage of the second reproductive cycle treated with EE_2_ or Tmx during a short term period and a short recovery time. The fish were treated with 0, 5 µg EE_2_ or 100 µg Tmx g^−1^ food during 15 and 30 days and after 6 and 22 days of reverting to the commercial diet. The peroxidase (**a**), bactericide (**b**), protease (**c**) and antiprotease (**d**) activities were studied. Data represent means ± standard error (n = 6). Different letters denote statistically significant differences between the groups (P < 0.05).

### Tmx and EE_2_ differently modifies the antimicrobial activities in SG fish of the second RC

Gilthead seabream in the SG stage of the second RC treated with either 5 µg EE_2_ g^−1^ food or 100 µg Tmx g^−1^ food showed scarce changes in some but not all antimicrobial activities compared to control fish (Fig. 3). Thus, peroxidase activity was sharply increased in fish fed with EE_2_ after 15 days of treatment but not in fish fed with Tmx (Fig. 3a). The fish treated with EE_2_ trended to recover similar levels to control fish after 30 days of treatment and reached control levels during the recovery period (Fig. 3a). Regarding bactericidal activity, only Tmx food intake inhibited bactericidal activity after 15 days of treatment, showed all experimental groups similar levels after 30 days of treatment and during the recovery time (Fig. 3b). No changes were found in protease (Fig. 3c) and antiprotease (Fig. 3d) activities of treated fish compared with control, although differences through time were observed in the antiprotease activity levels during the experiment (Fig. 3d and supplementary data).

**Figure 4:**
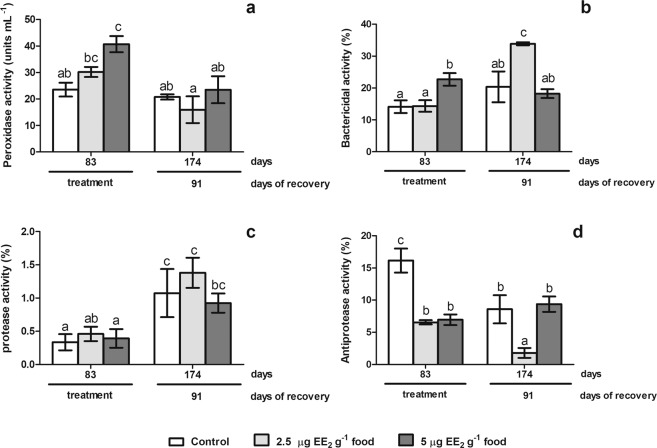
Antimicrobial humoral activities in gilthead seabream males in spermatogenesis stage of the second reproductive cycle treated with EE_2_ during a long term period and a long recovery time. The fish were treated with 0, 2.5 or 5 µg EE_2_ g^−1^ food for 83 days (from spermatogenesis stage to post-spawning stage) and after 91 days (testicular involution stage) of reverting to the commercial diet. The peroxidase (**a**), bactericide (**b**) protease (**c**) and antiprotease (**d**) activities were analyzed. Data represent means ± standard error (n = 6). Different letters denote statistically significant differences between the groups (P < 0.05).

### Long-term treatment with EE_2_ alters the antimicrobial functions in SG fish of the second RC

When fish at the SG stage of the second RC were fed with 2.5 or 5 µg EE_2_ g^−1^ food during 83 days (Fig. 4), the peroxidase (Fig. 4a) and the bactericidal (Fig. 4b) activity levels increased with the highest dose, while the antiprotease activity levels decreased with both doses (Fig. 4d). The protease activity levels remained unaltered upon both treatments (Fig. 4c).

**Figure 5:**
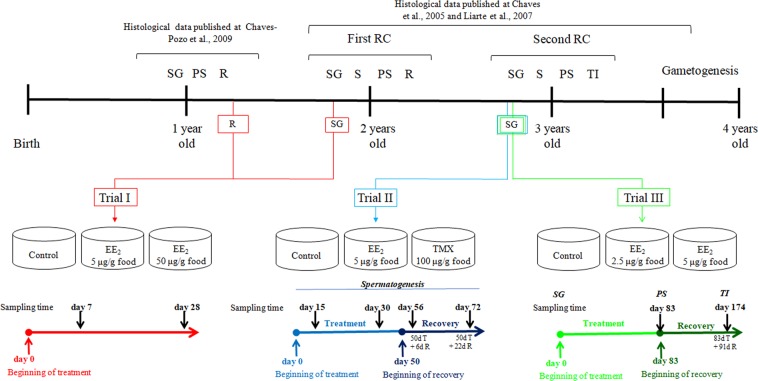
Schematic illustration of the timing of the different experiments related to the reproductive stages and age (years) of the gilthead seabream. RC, reproductive cycle; SG, spermatogenesis stage; S, spawning stage; PS, post-spawning stage; R, resting stage; TI, testicular involution stage; EE_2_, 17α-ethynilestradiol; Tmx, tamoxifen; dT, days of treatment; dR, days of recovery. Data obtained from this manuscript and from Chaves-Pozo *et al*. (2005, 2009)^24,39^ and Liarte *et al*. (2007)^25^.

During the recovery period, the peroxidase (Fig. 4a) and protease (Fig. 4c) activity levels did not show statistical differences between experimental groups. Strikingly, the bactericidal activity levels was increased (Fig. 4b) while the antiprotease activity levels decreased (Fig. 4d) in the fish treated with 2.5 µg EE_2_ g^−1^ food after 91 days of recovery compared to control fish levels. Differences in the same treated group through the time were observed in all the activities in control, as described previously, and in some treated groups (Fig. 4 and supplementary data).

## Discussion

Up to date, it is widely known that EDCs alters the functions, the levels or the body distribution of endogenous hormones in all vertebrates, including fish^36^. Between them, xeno-estrogens modify the estrogenic regulation of multiple biological process, including the immune response, whose seasonality in fish has been demonstrated^37,38^. The regulation of the immune response by estrogens in fish has been taken into consideration and the data demonstrate that both estrogens and estrogenic compounds alters the immune response of several fish species through genomic and non-genomic mechanisms of action^3,20,22^. Gilthead seabream is a hermaphroditic protandrous, seasonal breeder fish species that develop a testis during the first three years of life; however, as they are not able to spawn at the first year, it is only consider two consecutive mature RC of males: the first and second RC during the second and third year of life, respectively. Afterwards testicular involution and sex change takes place^25,39^ (Fig. 5). During the last two decades, different studies has reported the existence of hormonal receptors, including steroid sex hormone receptors, in different types of leukocytes and how leukocyte functions are regulated by them (see for review^20,22,40–42^). This leads to the hypothesis that the immune response might vary depending on the reproductive season of fish as sex hormones levels are modify depend on the reproductive season. Interestingly, some studies have analyzed some humoral activities through a part of the year, mainly the winter season, but none of them have related the differences observed with the reproductive stage of the fish. Our data showed, for the first time, in gilthead seabream that during the first and second RCs, the antimicrobial activities varied through the different reproductive stages. Thus, in the first RC, bactericidal and protease activities levels decreased as spermatogenesis processed, while in the second RC only the antiprotease activity levels progressively decreased through the spermatogenesis stage. Interestingly, the antiprotease activity levels further decreased at testicular involution stage compared to post-spawning stage that showed similar levels than those recorded at the end of the SG stage. In contrast, the protease activity slightly increased at testicular involution stage compared to post-spawning stage. So our data suggested that the humoral innate antimicrobial activities undergo slightly changes through the year related to the reproductive stage of the fish. In fact, the bactericide and peroxidase activity in European sea bass serum changed independently of the temperature through the year, suggesting this a relation between these humoral activities and the RC^38,43^, although more detailed studies will be needed to clearly relate this responses with the different reproductive stages and/or RCs.

It is worthy to note that the levels of protease activity recorded in these experiments are quite low (around 1% of activity) comparing with other published studies, although high variation was observed between published data (from 30 to 7%)^44–47^. The probable under detection due to methodological issues might be discarded as all the control fish of all the experiments reported in this study showed protease activity levels.

E_2_ has a key role in the male physiology in fish. In spermatogenetic gilthead seabream males, high doses of exogenous E_2_ during short period (18 days) or low doses during middle period (28–25 days) disrupted the spermatogenesis and triggered the post-spawning stage regulating the infiltration of acidophilic granulocytes, the main phagocytic cell, into the gonad^15,28^, but did not induce nor accelerate the natural sex change of gilthead seabream. However, in other hermaproditic fish species is has been reported that exogenous treatments with estrogens induce the change male to female (see for review^48^) as also occurs in gilthead seabream upon exposures to high doses of estrogens during long time^49^. All the trial reported in this manuscript were design to determine the effect of estrogens on male physiology and none of them triggered the sex change of the specimens.

Regarding leukocytes, E_2_ activate gilthead seabream macrophages than in turn recruited the acidophilic granulocytes and enhance the expression of adhesion molecules in the vascular epithelium enhancing the inflammatory response and the trafficking of leukocytes into tissues^26,33^. Estrogen receptor has been described in different types of leukocytes such as macrophages, neutrophils and lymphocytes and in different fish species (for review see^20,22^). In fact in gilthead seabream, EE_2_ has been described to affect the cellular and humoral specific immune response^2,30,31^. Moreover, the expression levels in the gonad of genes coding for antimicrobial peptides and pro- and anti-inflammatory cytokines show differences between the different stages of the RC in gilthead seabream and European sea bass^43,50^. Unfortunately, the effect of EDCs on the systemic humoral innate immune response and its correlation with the reproductive stage of the specimen has never been boarded in gilthead seabream. With this background, we have studied the effect on the antimicrobial humoral response of gilthead seabream males at different ages and exposure to two EDCs, EE_2_ or Tmx, with different estrogenic effects. In gilthead seabream, EE_2_ has a potent estrogenic effect as sharply increase the E_2_ serum levels and the transcription of the hepatic vitellogenin gene (*vtg*) as well as the transcription of the gonadal ER^15,16^. In mammals Tmx, acts as an estrogens agonist or antagonist depending on the target, highlighting the complexity of mechanisms mediating ER activities^11^. In gilthead seabream, the administrated Tmx dose has previously been described to be enough to ensure the Tmx-ER interactions^12^. In fact, in gilthead seabream males, 100 µg of Tmx g^-1^ of food, the dose used in this work, increased the E_2_ serum levels and the transcription of the hepatic *vtg* and the gonadal ER genes^12^. However, Tmx also increased the serum levels of androgens and the expression of genes involved in testicular development such as *dmrt1* gene^12^. Our results demonstrated that some humoral antimicrobial activities are sensitive to estrogenic compounds although we observed heavy differences between the effect of EE_2_ and Tmx as the latest only affect the bactericidal activity after 15 days of treatment but not after 30 days.

Peroxidase enzymes are involved in homeostasis, but they are also imperative microbicidal agents which effectively remove H_2_O_2_ produced during the respiratory burst process (between others) and preserve the redox balance of immune system^51^. In all fish studied (one and two years old fish), the peroxidase activity increased after short (7 or 15 days), middle (28 or 30 days) or long (83 days) treatments with doses of 5 µg EE_2_ g^−1^ food or higher. Similarly, increases in the peroxidase activity were observed when one-year old seabream specimens were treated with the endogenous estrogen, E_2_^52^. Despite the fact that Tmx dose was 20-fold higher than EE_2_ dose, this EDC did not increase the peroxidase activity during 30 days of treatment. Taking into account that EE_2_ is decomposed by a catalytic reaction triggered by peroxidases^53^. The possible role of peroxidases in the clearance of toxic levels of EE_2_ might explain why EE_2_ increase this activity but not Tmx. Thus, further studies should be conducted to undoubtedly establish the possible role of peroxidases in the control of xeno-estrogens in fish and how this process affects the antimicrobial responses of fish. In spite of this, the alteration of the immune responses based on the clearance and production of ROIs in which peroxidase activity is involved should not be undervalued as previous data obtained in gilthead seabream and based on *in vivo* studies determined that environmental concentrations of EE_2_ (50 ng mL^−1^) inhibit cell mediated innate immune activities such as ROIs production triggered by stimulation with bacterial DNA^30^. In the other hand, in gilthead seabream males treated with 5 µg EE_2_ g^−1^ food during 50 days the ROIs production triggered by an immune stimulus was inhibited during the treatment but was able to recover control values 6 days upon the cease of the exposure^2^. Moreover, *in vitro* treatments with EE_2_ in gilthead seabream leukocytes alter their ROIs production capability and their phagocytic activity^30^.

Concerning the bactericidal activity, short (7 days) and middle (28 days) exposures with doses of 50 µg EE_2_ g^−1^ food or long (83 days) exposures with lower doses (5 µg EE_2_ g^−1^ food) increased this activity. However, when middle (15, 28 or 30 days) exposures were applied, no differences in the bactericide activity were observed between treated and control fish at doses of 5 µg EE_2_ g^−1^ food. All these data together suggest that the bactericidal activity is affected by EE_2_ depending on dose, the time of exposure and the age of gilthead seabream males. Similarly, the total bactericidal activity of Japanese sea bass (*Lateolabrax japonicus*) also increased after middle bath exposures (30 days) with 200 or 2000 ng of E_2_ L^−1^ ^34^. However, no more studies have been carried out to study the effects of estrogenic compounds on bactericidal activity in fish. Nevertheless, in mammals, E_2_ exerts stimulatory effect on bactericidal activity even preventing sepsis^54,55^. Although further studies are mandatory, there are clear evidences pointing to a thigh relationship between estrogens and the immunocompetence against bacteria in vertebrates including fish.

Other antimicrobial activities, the protease and the antiprotease activities, have been extensively used as humoral innate immune indicators in gilthead seabream^44–47^. Our data showed that these activities were hardly altered by EE_2_ exposures. Thus, in one-year old fish at resting stage the antiprotease activity increased after 7 days of exposure with 5 µg EE_2_ g^−1^ food, while in fish at the subsequent spermatogenesis stage (at the first RC) the protease activity decreased after 7 days of 50 µg EE_2_ g^−1^ food exposure. Interestingly, in two-years old gilthead seabream males (second RC), neither the protease activity nor antiprotease activity were altered by EE_2_ after short or middle time exposures, while after 83 days of 2.5 or 5 µg EE_2_ g^−1^ food treatments the antiprotease activity was sharply inhibited and kept low after 91 days of the cease of the treatment in the fish fed with 2.5 µg EE_2_ g^−1^ food.

In general, our data show that most of the antimicrobial activities analysed were enhanced by the treatments. Similarly, some antimicrobial responses such as the production of ROIs and lysozyme activity increased in juveniles of yellow catfish (*Pelteobagrus fulvidraco*) subjected to EE_2_ bath exposure during 56 days^35^. Strikingly, our data are the first one to determine that only the antiprotease activity remained inhibited after 91 days of the cease of a long term exposure (83 days) with a low dose of EE_2_ (2.5 µg EE_2_ g^−1^ food). Several studies have pointed to the fact that environmental factors affect the immune response mainly upon a challenge state^30,56^. In contrast, our data demonstrate that low doses of estrogenic compounds unbalance the naïve humoral innate antimicrobial activity levels at certain point during the exposure or later on upon the cease of the exposure. Whether this effect might impair the immune response during a challenge is something that should be taken into consideration. However, all these data acquire an especial relevance when taking into account that there are fish pathogens which alter sex hormone levels to get profit of their regulatory role on the immune responses in an attempt to remain latent and/or spread^57^. Moreover, the transcription of genes involved in antiviral responses, are positively correlated with the expression levels of genes coding for ERs in gilthead seabream^57^. Indeed, most of the genes implied in the interferon I pathway are positively correlated with *era* gene in gilthead seabream upon a viral infection^57^. Remarkably, this receptor is expressed in gilthead seabream head kidney macrophages and lymphocytes^26^. These data suggest that estrogenic compounds would alter the capacity of gilthead seabream to respond upon viral infections although further investigations are needed to clarify this issue.

To summarize, the humoral innate immune activities analyzed showed different levels through different reproductive stages and ages in gilthead seabream. The EDCs, EE_2_ and Tmx, are found in fluent waters and are known to mimic estrogens provoking strong alterations in reproductive and immune functions in fish^2,3^. Gilthead seabream humoral innate immune activities are more sensitive to EE_2_ than to Tmx, which only inhibited the bactericidal activity after a short term exposure. However, the effects induced by EE_2_ vary depending on the activity analyzed, the dose and time of exposure and the reproductive stage and the age of the specimens. Strikingly, peroxidase activity is increased in all trials, but only upon EE_2_ treatment, probably as a way of decompose toxic level. Whether this issue might disturb the innate immune response should be taken into account for further studies. Interestingly, only the bactericidal and antiprotease activities showed unbalanced levels upon the cease of long term treatments with low doses of EE_2_. This also occurs with the humoral adaptive immune response of gilthead seabream but not with the cell-mediated innate immunity^2,31^. The long lasting effects, even when being scarce, upon the cease of the treatment observed in gilthead seabream in this work and other^2^, lead to the need to develop proper wastewater treatment that definitively decompose these pollutants and release clean fluent waters in order to dismiss their possible effect on aquatic organism populations.

## Material and Methods

### Experimental procedures

#### Animals

Healthy specimens of gilthead seabream (*Sparus aurata*, L.) were bred and kept at the *Centro Oceanográfico de Murcia* (*Instituto Español de Oceanografía*, Mazarrón, Murcia, Spain). The fish were kept in 2 m^3^ tanks with a flow-through circuit, suitable aeration and filtration system and natural photoperiod. The water temperature ranged from 14.6 to 17.8 °C. The environmental parameters, mortality and food intake were recorded daily.

#### Diets preparation, administration and sampling

All experiments were conducted by adding EE_2_ (purity 98%; Sigma) or Tmx (Sigma) to a commercial diet (44% protein, 22% lipids, Skretting), which was used as control. Thus, the EE_2_ was incorporated in the commercial food at doses of 0 (control), 2.5, 5 or 50 μg g^−1^ food, and Tmx at 100 μg g^−1^ food using the ethanol evaporation method (0.3 L ethanol/kg of food) as described elsewhere^58^. In all cases the specimens were fed *ad libitum* three times a day and fasted for 24 h before sampling. Prior to fish handle, all specimens (n = 6 fish/group/sampling time) were anesthetized with 40 µl/L of clove oil in seawater, completely bled and immediately beheaded and weighed. Serum samples were obtained by centrifugation from trunk blood collection (10,000 xg 10 min 4 °C) and immediately frozen and stored at – 80 °C until use.

#### Experimental designs

Three different trials were performed (Fig. 5):

- Trial I. Using 36 gilthead seabream males at resting (R) stage previous to the beginning of the first RC (the stage just before to the spermatogenesis stage; 110 ± 20 g of body weight, bw; 14-months old) and 36 at spermatogenesis (SG) stage of the first RC (405 ± 25 g of bw; 19 months old). The specimens were fed during 28 days (treatment period) with a diet supplemented with EE_2_ at the doses of 0 (Control), 5 or 50 μg of EE_2_ g^−1^ food and sampled at days 7 and 28 of EE_2_ exposure.

- Trial II. Using 72 gilthead seabream males in SG stage of the second reproductive cycle (453 ± 7.0 g bw) orally exposed to 0 (Control), 5 μg EE_2_ g^−1^ food or 100 μg Tmx g^−1^ food during 50 days (treatment period) and then fed with commercial diet (recovery period) during 22 days more. All the trial was performed during the spermatogenesis stage of the specimens. Sampling was performed at 15 and 30 days of exposure (days of treatments) and at days 6 and 22 of the recovery period (days 56 and 72 of the trial, respectively).

- Trial III. With 54 gilthead seabream males at SG stage of the second reproductive cycle (166 ± 24 g bw). We exposed the fish with 0 (Control), 2.5 or 5 μg of EE_2_ g^−1^ food during 83 days (treatment period). After the treatment period, specimens were fed with a commercial diet during 91 additional days (recovery period). Sampling was performed at the end of the treatment period (83 days of the beginning of the trial that corresponded with post-spawning stage) and at day 91 of the recovery period (day 174 of the beginning of the trial that corresponded with testicular involution stage).

### Antimicrobial humoral activities

The peroxidase activity in serum was measured according to a previously described protocol^59,60^. One unit was defined as the amount of activity producing an absorbance change of 1 and the activity was expressed as units mL^-1^ serum.

The bactericidal activity of serum was determined by evaluating their effects on the bacterial growth of *V. harvevi* curves as elsewhere^59,61^. The pathogenic marine bacteria *Vibrio harveyi* (strain Lg 16/100) was grown and cultured as previously described^59^. Results were corrected with the absorbance measured in each sample at the initial time point and expressed as % of activity in serum.

The protease activity in serum was determined as the percentage of hydrolysis of azocasein using a modified formerly defined protocol^62^ and briefly described elsewhere^59^. The percentage of protease activity for each sample was calculated as % of the activity referred to the positive control (10 μL of 2 mg/mL proteinase K (AppliChem) in PBS replaced the sample). Results were expressed as % of activity in serum.

The antiprotease activity was determined by the ability of serum to inhibit proteinase K activity using a modified previously described protocol^63^ and briefly described elsewhere^59^. The percentage of inhibition of proteinase K activity for each sample was calculated as [100-(% of protease activity)]. Results were expressed as % of activity in serum.

### Statistics

All data are presented as mean ± standard error of the mean (SEM) and the statistical analysis performed as described elsewhere^59^. The data were subjected to a Shapiro-Wilk test to determine their normality and to a Levene test to verify the homogeneity of variances. Then they were analysed by one- way ANOVA followed by a LSD (Fisher Least Significant Difference) *post hoc* test to denote statistical differences between groups and by two-way ANOVA to determine differences between sampling times. When some parameters did not meet normally assumptions, the data were log-transformed prior to analysis or a non-parametric Kruskal–Wallis test, followed by a multiple comparison test, was applied. Statistical analyses were conducted using STATGRAPHICS Centurion XV 15.2.06 software. Significance level (P) was fixed at 0.05.

### Ethical approval

All specimens studied were handled in accordance with the Guidelines of the European Union Council (2010/63/UE), the Bioethical Committees of the IEO (reference REGA ES300261040017) and the “Consejería de Agua, Agricultura y Medio Ambiente” of the “Región de Murcia”, Spain (approval number A13160507).

## Supplementary information

Supplementary Information.

## Data Availability

All data are available upon request, please contact Dr. Elena Chaves-Pozo (email address: elena.chaves@ieo.es).
