# Supplementary material for: Endocrine disrupter chemicals affect the humoral antimicrobial activities of gilthead seabream males even upon the cease of the exposure
Source: Sci Rep. 2020 May 14;10:7966. doi: 10.1038/s41598-020-64522-2 (PMC7224181; doi:10.1038/s41598-020-64522-2)
Supplement: Supplementary file 1 — Supplementary Information. [file 41598_2020_64522_MOESM1_ESM.docx]

**Endocrine disrupter chemicals affect the humoral antimicrobial activities of gilthead seabream males even upon the cease of the exposure**

Yulema Valero^1^, Amanda E. López-Cánovas^2^, M. Carmen Rodenas^2^, Isabel Cabas^2^, Pilar García-Hernández^2^, Marta Arizcun^1^, Alfonsa García-Ayala^2^, Elena Chaves-Pozo^1*^

**Supplementary data:**

|  | **Activity** | **Comparison type** | **F value** | **Significance^1^** |
| --- | --- | --- | --- | --- |
| Trial II | Peroxidase | Treatment | 20.066 | **0.000** |
|  |  | Time | 10.024 | **0.000** |
|  |  | Treatment*Time | 10.171 | **0.000** |
|  | Bactericidal | Treatment | 6.24 | **0.003** |
|  |  | Time | 1.81 | 0.156 |
|  |  | Treatment*Time | 1.54 | 0.183 |
|  | Protease | Treatment | 2.34 | 0.107 |
|  |  | Time | 0.51 | 0.677 |
|  |  | Treatment*Time | 0.87 | 0.526 |
|  | Antiprotease | Treatment | 2.7 | 0.078 |
|  |  | Time | 26.07 | **0.000** |
|  |  | Treatment*Time | 1.1 | 0.373 |
| Trial III | Peroxidase | Treatment | 10.09 | **0.000** |
|  |  | Time | 50.25 | **0.000** |
|  |  | Treatment*Time | 1.74 | 0.165 |
|  | Bactericidal | Treatment | 1.94 | 0.159 |
|  |  | Time | 23.65 | **0.000** |
|  |  | Treatment*Time | 6.38 | **0.000** |
|  | Protease | Treatment | 2.22 | 0.125 |
|  |  | Time | 6.77 | **0.004** |
|  |  | Treatment*Time | 0.4 | 0.808 |
|  | Antiprotease | Treatment | 23.35 | **0.000** |
|  |  | Time | 5.54 | **0.008** |
|  |  | Treatment*Time | 4.9 | **0.003** |

^1^Statistical limit established in P<0.05.

Statistical significances marked in bold.
